# Toward h-BN/GaN Schottky Diodes: Spectroscopic Study on the Electronic Phenomena at the Interface

**DOI:** 10.1021/acsami.1c20352

**Published:** 2022-01-19

**Authors:** Ewelina Zdanowicz, Artur P. Herman, Katarzyna Opołczyńska, Sandeep Gorantla, Wojciech Olszewski, Jarosław Serafińczuk, Detlef Hommel, Robert Kudrawiec

**Affiliations:** † Łukasiewicz Research Network—PORT Polish Center for Technology Development, Stabłowicka 147, Wrocław 54-066, Poland; ‡ Department of Semiconductor Materials Engineering, Wrocław University of Science and Technology, Wyspiańskiego 27, Wrocław 50-370, Poland; § Institute of Experimental Physics, University of Wrocław, pl. M. Borna 9, Wrocław 50-204, Poland; # Department of Nanometrology, Wrocław University of Science and Technology, Janiszewskiego 11/17, Wrocław 50-372, Poland

**Keywords:** h-BN, GaN, surface potential barrier, Fermi level pinning, Schottky diode, contactless electroreflectance

## Abstract

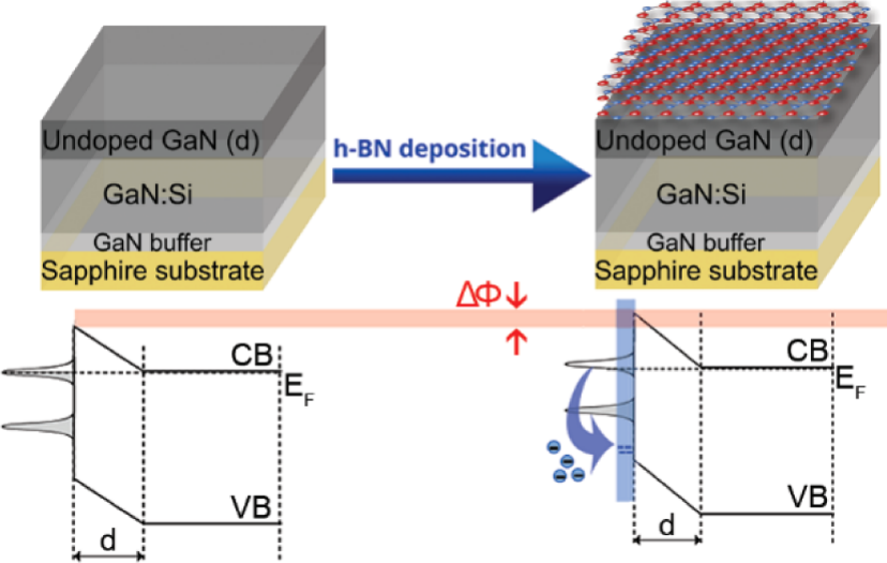

Hexagonal boron nitride (h-BN), together with other members of the van der Waals crystal family, has been studied for over a decade, both in terms of fundamental and applied research. Up to now, the spectrum of h-BN-based devices has broadened significantly, and systems containing the h-BN/III-V junctions have gained substantial interest as building blocks in, inter alia, light emitters, photodetectors, or transistor structures. Therefore, the understanding of electronic phenomena at the h-BN/III-V interfaces becomes a question of high importance regarding device engineering. In this study, we present the investigation of electronic phenomena at the h-BN/GaN interface by means of contactless electroreflectance (CER) spectroscopy. This nondestructive method enables precise determination of the Fermi level position at the h-BN/GaN interface and the investigation of carrier transport across the interface. CER results showed that h-BN induces an enlargement of the surface barrier height at the GaN surface. Such an effect translates to Fermi level pinning deeper inside the GaN band gap. As an explanation, we propose a mechanism based on electron transfer from GaN surface states to the native acceptor states in h-BN. We reinforced our findings by thorough structural characterization and demonstration of the h-BN/GaN Schottky diode. The surface barriers obtained from CER (0.60 ± 0.09 eV for GaN and 0.91 ± 0.12 eV for h-BN/GaN) and electrical measurements are consistent within the experimental accuracy, proving that CER is an excellent tool for interfacial studies of 2D/III–V hybrids.

## Introduction

The graphitic hexagonal polymorph of boron nitride (h-BN) has been investigated for over a decade.^1^ Nevertheless, it was the graphene discovery^2^ in 2004 that made the research interest in h-BN and other van der Waals crystals truly skyrocketing. Shortly after the initial studies oriented toward the applications of individual ‘wonder’ two-dimensional (2D) nanomaterials, the efforts to combine their unique properties catapulted. In this context, the most important characteristics of white graphene (h-BN), to name a few, are its chemical stability, thermal conductivity, and wide band gap (5.1–5.9 eV).^3,4^ All of them made h-BN the material of choice in various 2D stacked devices as an encapsulating layer or a substrate.^5^ Obviously, in parallel, combinations of h-BN with technologically mature materials like Si, GaAs, or GaN have also appeared. Examples include a graphene/h-BN/n-Si heterojunction photovoltaic cell,^6^ cubic-BN/h-BN/Si heterojunction^7^ or graphene/h-BN/GaAs solar cell, and photodetector.^8^ Pairing h-BN with GaN resulted in the development of, inter alia, light emitting diodes (LEDs),^9−11^ UV photodetectors,^12,13^ and metal–insulator–semiconductor high-electron mobility transistors (MISHEMTs).^14^ In the case of GaN-based light emitters, h-BN provides electron blocking and more efficient hole injection into the active region. For transistor structures, h-BN acts as a passivation layer and gate dielectric, neutralizing the surface traps and reducing the leakage current. In photodetectors, it helps to decrease the dark current.^12,13^

The Fermi level position at the heterostructure’s interface is a crucial issue for the device’s operation because the latter may be influenced by the surface-related phenomena. Specifically, the surface Fermi level position governs the incorporation of impurities and defects during the growth;^15^ it influences the surface’s barrier height in the case of metal–semiconductor junctions, which in turn translates to the type of electrical contact,^16,17^ and it is also a boundary condition for the distribution of polarization-related fields inside the HEMT structures.^18^

The h-BN/GaN interface represents the interface of the van der Waals crystal and III–V semiconductor. Bearing in mind the potential of h-BN/III–V-based devices together with the significance of surface-related phenomena, the study of the surface barrier height on the h-BN/GaN junction becomes indispensable, but it has not been proposed yet. In this work, we probed built-in electric field in h-BN/GaN structures using contactless electroreflectance (CER) in order to investigate the carrier transport across the h-BN/GaN interface. The physical explanation of the origin of Fermi level pinning at such an interface is proposed for the first time.

## Materials and Methods

### GaN Growth

GaN van Hoof structures were grown via Metal-Organic Vapor Phase Epitaxy (MOVPE) using a vertical reactor (CCS3x2FT AIXTRON) on 430 μm c-plane sapphire substrates with a 0.2° offcut. Trimethylgallium and ammonia (NH_3_) were used as precursors, and hydrogen (H_2_) was used as a carrier gas. Structures consisted of a 1.5 μm undoped GaN buffer layer, 0.5 μm n-type GaN layers doped with silicon using silane (SiH_4_) with a dopant concentration level of 5.5 × 10^18^ cm^–3^, and a capping layer of 20/50/80 nm undoped GaN. The thickness of the cap was established based on our standard growth procedures.^19^ GaN layers were grown at a pressure of 150 mbar and at a temperature of 1045 °C.

### h-BN Transfer

(1) Prior to the transfer of h-BN van Hoof, GaN supports were soaked in a mixture (1:1 v/v) of concentrated HCl_aq_ (Chempur) and methanol (HPLC-grade, Sigma Aldrich) for 5 min under an argon atmosphere; (2) Poly (methyl methacrylate) (PMMA)-assisted transfer—general procedure: h-BN on a Cu foil (2D semiconductors) was covered with a polymer [5 wt % solution of PMMA (*M*_W_ 350,000, Sigma-Aldrich) in anisole (Merck)] using spin coating (POLOS, Spin150). The as-prepared material was subsequently annealed under an argon atmosphere for 1 min at 100 °C. Then, the copper substrate was etched using a 0.2 M aqueous solution of FeCl_3_. The remaining h-BN/PMMA was washed thoroughly with deionized (DI) water and transferred to the target van Hoof GaN structure. The resulting PMMA/h-BN/GaN structure was annealed in air at 300 °C to a constant weight in order to burn the polymeric residue.

### S/TEM Characterization

The cross-sectional transmission electron microscopy (TEM) specimen lamella was prepared by a standard focused ion-beam (FIB) milling technique using an FEI Helios NanoLab H50HP SEM/FIB microscope equipped with Ga+ ion gun. Prior to TEM sample preparation, the sample was coated with a ∼30 nm thick amorphous carbon film (protection layer) by carbon sputtering. S/TEM characterization was performed on the Thermo Fisher Scientific Titan 60–300 cubed. S/TEM is equipped with a high brightness X-FEG gun, a Wien filter monochromator, an image Cs-corrector, a DCOR probe Cs-corrector, a ChemiSTEM super-X EDS 4-detectors system, and a Gatan continuum EELS spectrometer, using the 80 kV operating voltage. Conventional TEM and high-resolution TEM (HRTEM) imaging were carried out to investigate the cross section of the sample. Electron energy loss spectroscopy (EELS) characterization of the specimen was carried out in the scanning transmission electron microscopy mode (STEM) using the annular bright field imaging technique (ABF). STEM-ABF imaging was performed with a probe current of ∼100 pA, the probe beam convergence angle was 21.4 mrad, and the ABF detector collection angle was in the range of 10–19 mrad.

### X-Ray Diffraction (XRD) Characterization

The h-BN/GaN hybrids were investigated using an Empyrean X-ray diffractometer supported by a Pixcel three-dimensional (3D) detector in the Bragg–Brentano configuration and Cu_kα1_ = 1.540597 Å wavelength. The Bartels monochromator was used to improve the shape of the beam and increase the sensitivity of the measurement. The PDF-4 cards with no. 01-079-6757 for h-BN were used.

### Electrical Measurements and Processing of the h-BN/GaN Schottky Diode

A Schottky diode was fabricated using the van Hoof structure with a 50 nm undoped GaN cap (u-GaN). Firstly, mesa-area was etched in the ICP-RIE plasma etcher using a BCl_3_/Cl_2_/Ar mixture. After that, the h-BN layer was transferred onto the remaining u-GaN surface. Ni/Au and Ti/Al metals were applied for the Schottky contact and ohmic contact, respectively. The contacts were deposited using the physical vapor deposition method in 2 × 10^–7^ mbar pressure. The Schottky diode without the h-BN layer (reference) was prepared following the same procedure. After processing, the current–voltage (I–V) characteristics for the h-BN/GaN Schottky diode and the GaN Schottky diode were measured.

### CER Measurements

During the CER experiment, the sample was placed in the in-house designed capacitor in the following manner: the sample was attached (glued with a silver paste) to the bottom electrode, while the semitransparent upper one (made of copper wire mesh) was placed 0.5 mm above the sample. An alternating external voltage (280 Hz, 3 kV) provided the band-bending modulation. A single grating 0.75 m Andor monochromator dispersed the laser-driven xenon lamp light. The measurements were performed using the so-called dark configuration;^20^ that is, the sample was illuminated using the monochromatic light while the external modulation was on. Light reflected from the sample was detected by a lock-in technique using a photomultiplier. More details regarding the CER method together with a capacitor and experimental setup schemes can be found elsewhere.^20,21^

## Results and Discussion

### Samples Preparation

**Figure 1:**
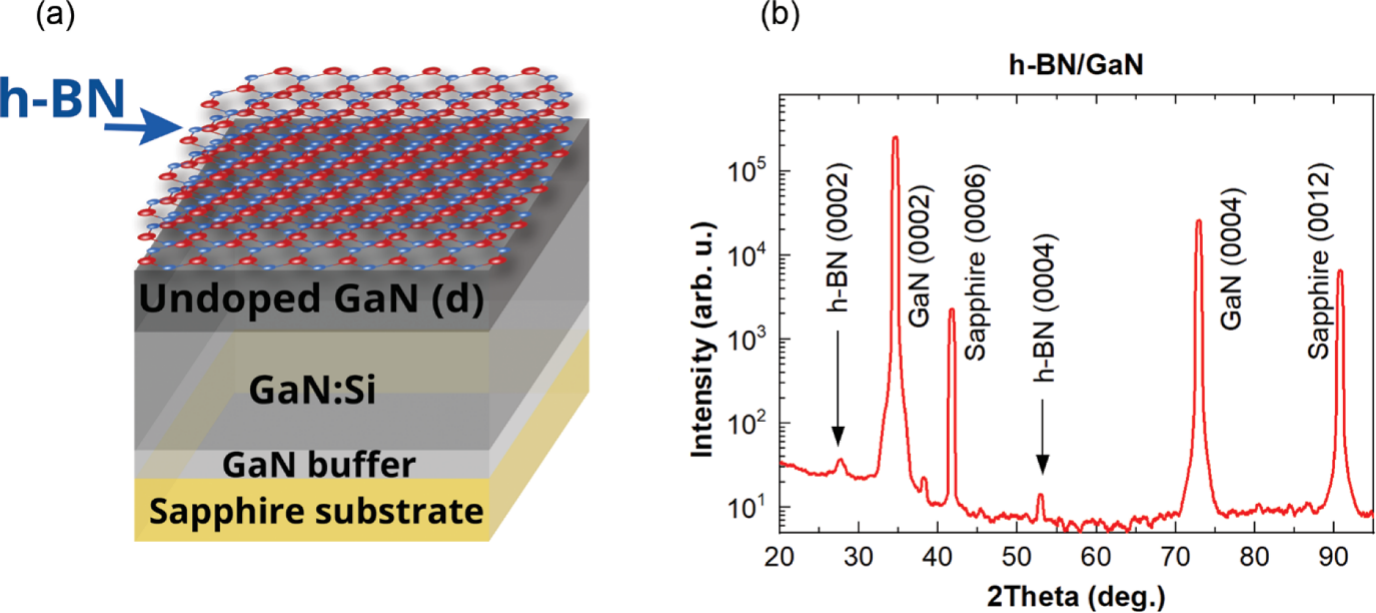
(a) General scheme of the h-BN/GaN structure. (b) Diffraction spectrum of the h-BN/GaN hybrid presented in (a).

In order to take advantage of the possibilities offered using modulation spectroscopy (particularly CER) and to fully understand the electronic phenomena at the h-BN/GaN interface, we grew the so-called van Hoof GaN structures. These are layered structures, where on a substrate (sapphire) followed by buffer layer (GaN), a highly doped layer (GaN:Si) is grown, and then capped with a nominally undoped layer (GaN) of a known thickness.^22^ The scheme of the GaN van Hoof structure covered with h-BN is presented in Figure 1a. The rationale behind the use of such structures is discussed thoroughly in the section devoted to CER measurements. The h-BN/GaN structures were prepared using commercially available h-BN (Cu substrate) via a well-known PMMA-assisted transfer method.

### Structural Analysis (XRD)

Figure 1b shows the diffraction spectra of the h-BN/GaN structure. Strong (0002) and (0004) reflections from GaN together with (0006) and (0012) from sapphire dominate the spectra; however, (0002) and (0004) reflections ascribed to h-BN^23^ can be clearly observed. The noticeably lower intensity of peaks assigned to h-BN when compared to GaN or sapphire results from the h-BN layer’s small thickness, as confirmed by using TEM (discussed below). The h-BN lattice parameter along the c direction of (0.6911 ± 0.0007) nm was determined using the Bragg equation from the (0004) reflection position.

### Morphological Analysis (S/TEM)

**Figure 2:**
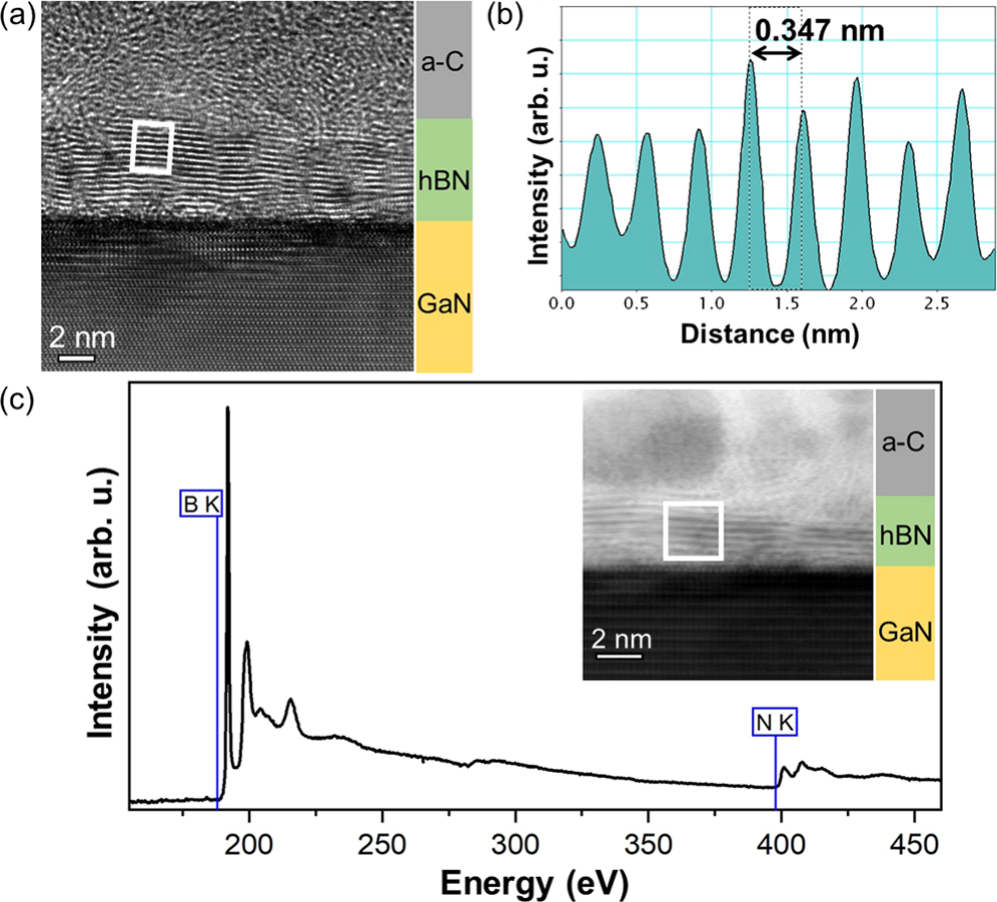
S/TEM characterization of the sample cross section. (a) HRTEM image showing the presence of a few layers of h-BN above GaN, amorphous carbon (aC) above the h-BN layers corresponds to the protection layer deposited for TEM specimen preparation. (b) Image intensity profile across the h-BN layers, corresponding to the white rectangle marker region in (a), showing their interlayer spacing. (c) EELS core-loss spectrum from the white rectangle marker region in the inset STEM-ABF image.

Figure 2a shows the HRTEM image, clearly revealing the presence of a few layers of h-BN observed in cross-sectional view above GaN. The number of h-BN layers along the GaN varied in the range of 7–20. Figure 2b shows the intensity profile across the h-BN layers from the white rectangle marker region in Figure 2a. The distance between the peaks corresponds to the interlayer spacing between the h-BN layers. The measured interlayer distance of 0.347 nm is consistent with the XRD measurements (0.346 nm) and within the acceptable experimental error (5%) of the bulk h-BN interlayer spacing of 0.333 nm.^24^ In order to evaluate the chemical composition of the as-transferred layers, EELS core-loss analysis was performed in the STEM-ABF imaging mode. Figure 2c shows the EELS spectrum from the white rectangle marker region in the inset image. The Boron-K and Nitrogen-K EELS edges can be clearly observed in the spectrum. This further confirms chemically that the transferred layers are indeed BN.

### Investigation of the h-BN/GaN Interface

CER is a representative of modulation spectroscopy techniques that require an external perturbation for the modulation of a chosen parameter inside the semiconductor material or structure. In the case of CER, an external electric field provides the band-bending modulation in the near surface area, which states the principle of operation. As a consequence, the dielectric function is perturbed by the electric field-induced band bending, leading to the appearance of resonant-like features in the modulated reflectance spectrum around energies corresponding to the energies of optical transitions. If a built-in electric field is present inside the structure, it gives rise to the Franz–Keldysh oscillations (FKO) visible above the fundamental transition of the investigated structure. The period of FKO is related to the strength of this field.^25,26^

At the doped/undoped GaN interface in the bare van Hoof structure, the Fermi level is located close to the conduction band edge (CBE) due to the n-type doping. On the other side of the undoped layer, the surface Fermi level position is pinned by the surface states to one of two characteristic for GaN surface densities of states (SDOS) present inside the GaN band gap.^21,27^ These SDOS are the consequence of the surface reconstruction and the presence of Ga dangling bonds on the GaN surface. The difference between the Fermi level position introduces a uniform electric field in the top, undoped layer of the van Hoof structure. The built-in electric field gives rise to FKO appearing in the CER spectra. Although GaN is covered with h-BN, the electronic passivation of the GaN surface states may occur, thus influencing the surface Fermi level position. Hence, the FKO analysis for bare and h-BN-covered van Hoof GaN structures gives the information about the electronic phenomena at the interface. The surface potential barrier can be calculated according to the analysis below. The asymptotic expression for electroreflectance^25^ (eq 1) describes the relationship between the FKO and the built-in electric field:
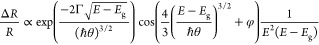
1
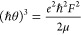
2where *ℏ*θ is the electro-optic energy, Γ is the linewidth, φ is the phase, *F* is the electric field, and μ is the electron–hole reduced mass for GaN. The FKO extrema are given by the formula:
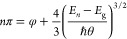
3where *n* is the index of the *n*-th extremum and *E*_*n*_ is the corresponding energy. A plot of (*E*_*n*_ – *E*_g_)^3/2^ versus *n* yields a straight line with a slope proportional to *F*. For the n-type structures,^21^ the relation between the electric field intensity *F* and the surface potential barrier Φ is given by:
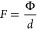
4where *d* is the thickness of the cap layer in the van Hoof structure because a homogenous electric field distribution is expected. In order to determine the Fermi level position at the surface, the obtained values of the electric field can be plotted as a function of *d* and fitted using eq 4 with the potential barrier Φ treated as a free parameter.

**Figure 3:**
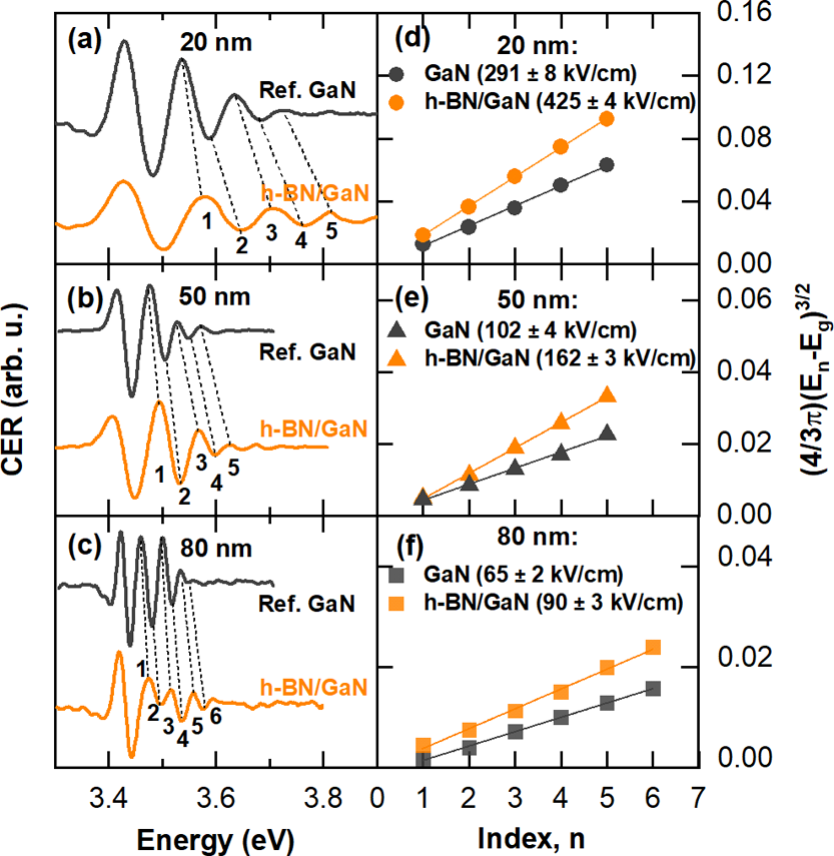
(a–c) Room temperature CER spectra of reference GaN and h-BN/GaN heterostructures for van Hoof structures with 20, 50, and 80 nm thick cap layers, respectively. The h-BN-induced change in the FKO period can be noticed and is indicated by eye-guiding lines. (d–f) Analysis of the built-in electric field for reference GaN and h-BN/GaN structures with 20, 50, and 80 nm thick GaN cap layers, respectively. Extracted values of the built-in electric field are given in the legend.

**Figure 4:**
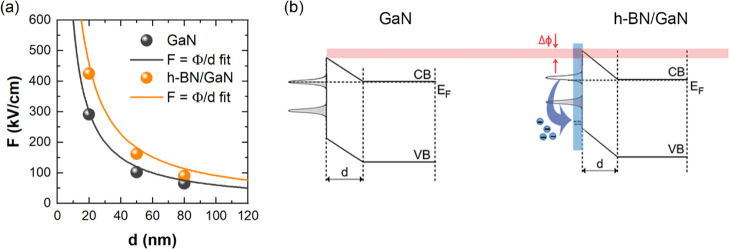
(a) Determination of surface barrier height for GaN and h-BN/GaN structures together with the fitting curves. Values of (0.60 ± 0.09) and (0.91 ± 0.12) eV for the GaN surface and h-BN/GaN interface were established, respectively. Experimental errors for *F* are smaller than the size of the points. (b) Schematic representation of h-BN-induced downward shift of the surface Fermi level position at the h-BN/GaN interface.

Van Hoof GaN structures with 20, 50, and 80 nm thick cap layers were investigated in our study. Figure 3a–c shows the room temperature CER spectra for reference GaN and h-BN/GaN structures. All spectra consist of GaN band gap-related resonance (3.43 eV) followed by the FKO (numbered). Assuming the same doping level in all GaN supports, a decrease in the value of the built-in electric field for thicker cap layers is expected.^28^ It is manifested as a shortening of the FKO period. Comparing the CER spectra, it can be easily noticed that afterh-BN coverage, the FKO period widens for each cap thickness. It indicates the increase in the built-in electric field in the h-BN/GaN hybrid induced by the h-BN transfer. The values of the electric field were calculated from the FKO period following eq 3, and they are depicted in the legend in Figure 3d–f. Experimental uncertainties were calculated according to the combined standard uncertainty formula. The increase in the values of built-in fields for h-BN/GaN structures indicates the increase in the surface barrier height for electrons inside these structures. In order to determine the barrier height for GaN and h-BN/GaN, the calculated values of the electric field have been fitted by formula 4 and plotted in Figure 4a. The values of (0.60 ± 0.09) and (0.91 ± 0.12) eV for GaN and h-BN/GaN were obtained, respectively. The value derived for GaN agrees reasonably with values reported for n-type GaN.^21,27,29^ Once the GaN surface was covered with h-BN, the surface Fermi level position had significantly changed, that is, it had moved deeper into the GaN band gap resulting in the increase of the surface barrier, which is schematically presented in Figure 4b. Here, it is worth to note that h-BN was reported to be intrinsically p-type.^30−32^ Recent theoretical works showed that intrinsic point defects^33^ such as nitrogen vacancy V_N_, boron antisite B_N_, and nitrogen interstitial N_i_ (or their complexes),^34^ and impurities such as substitutional carbon C_N_ and complexes with hydrogen V_B_–H can act as stable acceptors. Hence, we identified the transfer of electrons from the GaN surface states to the h-BN acceptor states as the main mechanism responsible for the increase in the surface barrier. Another reason responsible for the change of the potential barrier could be the change in the electronic state on the h-BN/GaN interface.

h-BN/GaN is an interface between a van der Waals crystal and a covalent crystal with a very significant mismatch between the lattice constants (*a*_h-BN_ = 2.502^35^ vs *a*_GaN_ = 3.189^36^ Å). Surface reconstruction is rather unexpected for van der Waals crystals, while it is known to occur in GaN. This reconstruction should remain unchanged after the transfer of h-BN. Nevertheless, annealing of the h-BN/GaN structure during the transfer process may result in an alteration in the reconstruction of the GaN surface. Hence, one cannot rule out the emergence of additional states at the h-BN/GaN interface, which can also cause an increase in the surface barrier height. However, even in this case, electrons from the h-BN/GaN interface will be captured by acceptor states in h-BN.

### h-BN/GaN Schottky Diode

**Figure 5:**
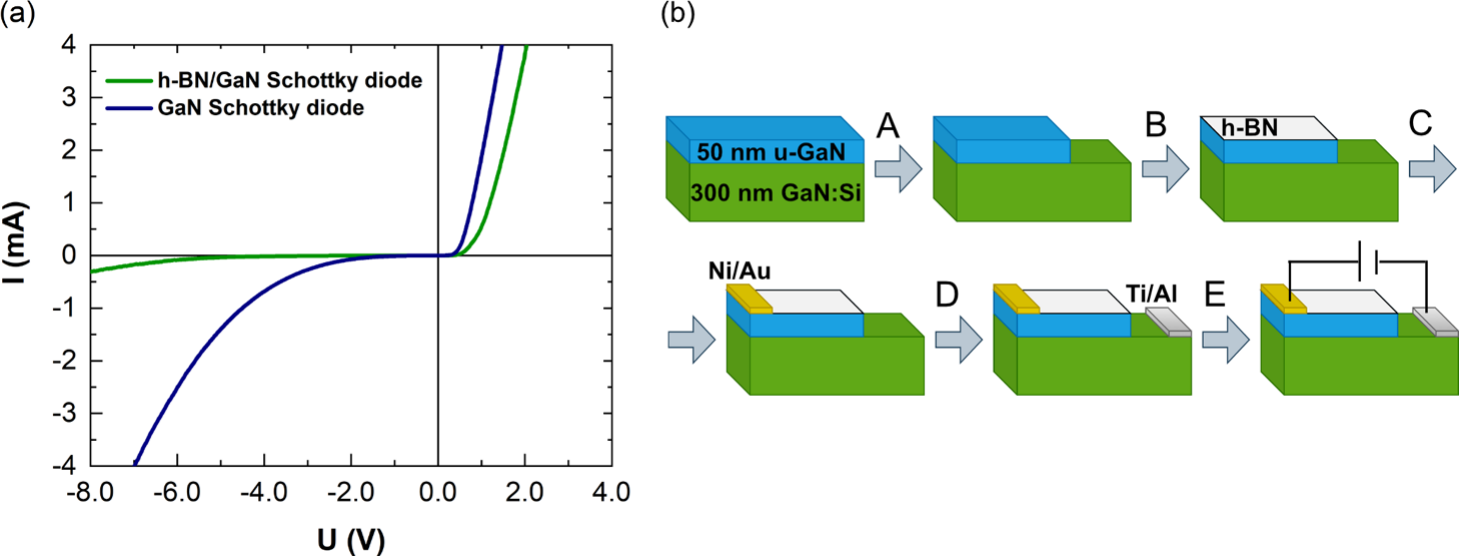
(a) Room temperature *I*–*V* characteristics of h-BN/GaN and reference GaN Schottky diodes. (b) Schottky diode processing steps: A—mesa etching, B—h-BN transfer, C—Schottky contact preparation, D −ohmic contact preparation, and E—electrical characterization.

In order to validate the CER results and examine the h-BN/GaN potential barrier height, the h-BN/GaN Schottky diode together with the reference GaN Schottky diode was fabricated. Figure 5a presents room temperature *I*–*V* characteristics of the investigated structures. In Figure 5b, the h-BN/GaN Schottky diode processing is shown.

As it can be seen from the *I*–*V* characteristics, the transfer of h-BN onto GaN support results in an increase in the forward voltage and a reduction of reverse current, indicating the increase of the potential barrier. Its height was estimated from the extrapolation of the linear part of the characteristics in the forward current region to the voltage axis. The values of 0.56 eV for bare GaN and 0.95 eV for the h-BN-containing structure were obtained. These values are consistent with surface barriers obtained from the CER experiment (0.60 ± 0.09 eV for GaN and 0.91 ± 0.12 eV for h-BN/GaN). More details concerning the electrical measurements accompanied by the temperature dependent *I*–*V* characteristics (Figures S1,S2) of both the reference GaN and the h-BN/GaN Schottky diode can be found in the Supporting Information.

## Conclusions

In this work, the electronic phenomena at the h-BN/GaN interface were investigated using CER spectroscopy. The latter enables nondestructive probing of the Fermi level position at the h-BN/GaN interface. The proposed methodology is fully applicable to other systems containing 2D nanomaterials (in particular van der Waals crystals) and bulk (covalent) crystals. Such hybrids open up new perspectives in barrier engineering regarding (among others) Schottky diodes. The transfer of h-BN onto the GaN support resulted in an electron shift from the GaN surface to h-BN acceptor states. This effect was manifested by the enlargement of the potential barrier observed in CER spectra. Importantly, the results were consistent with the *I*–*V* measurements of corresponding h-BN/GaN Schottky diodes.

## Supporting Information Available

Temperature-dependent *I*–*V* measurements of GaN and h-BN/GaN Schottky diodes; extended XRD diffraction analysis; and CER analysis of samples at different stages of the h-BN transfer process (PDF)

## Abbreviations

CER: contactless electroreflectance
MISHEMT: metal–insulator–semiconductor high-electron mobility transistor
FKO: Franz–Keldysh oscillations
CBE: conduction band edge
SDOS: surface density of states
