# Supplementary material for: Toward h-BN/GaN Schottky Diodes: Spectroscopic Study on the Electronic Phenomena at the Interface
Source: ACS Appl Mater Interfaces. 2022 Jan 19;14(4):6131–7. doi: 10.1021/acsami.1c20352 (PMC8815035; doi:10.1021/acsami.1c20352)
Supplement: Supplementary file 1 — am1c20352_si_001.pdf [file am1c20352_si_001.pdf]

Supporting Information for

# *Towards h-BN/GaN Schottky diodes: spectroscopic study on the electronic phenomena at the interface*

*Ewelina Zdanowicz,*[*1,2] Artur P. Herman,*[2] Katarzyna Opołczyńska,*[1,3] Sandeep Gorantla,*[1] Wojciech Olszewski,*[1] Jarosław Serafińczuk,*[1,4] Detlef Hommel,*[1] Robert Kudrawiec*[*1,2]

[1]Łukasiewicz Research Network – PORT Polish Center for Technology Development, Stabłowicka 147, Wrocław 54-066, Poland

[2]Department of Semiconductor Materials Engineering, Wrocław University of Science and Technology, Wyspiańskiego 27, Wrocław 50-370, Poland

[3]Institute of Experimental Physics, University of Wrocław, pl. M. Borna 9, Wrocław 50-204, Poland

[4]Department of Nanometrology, Wrocław University of Science and Technology, Janiszewskiego 11/17, Wrocław 50-372, Poland

*ewelina.zdanowicz@pwr.edu.pl

*robert.kudrawiec@pwr.edu.pl

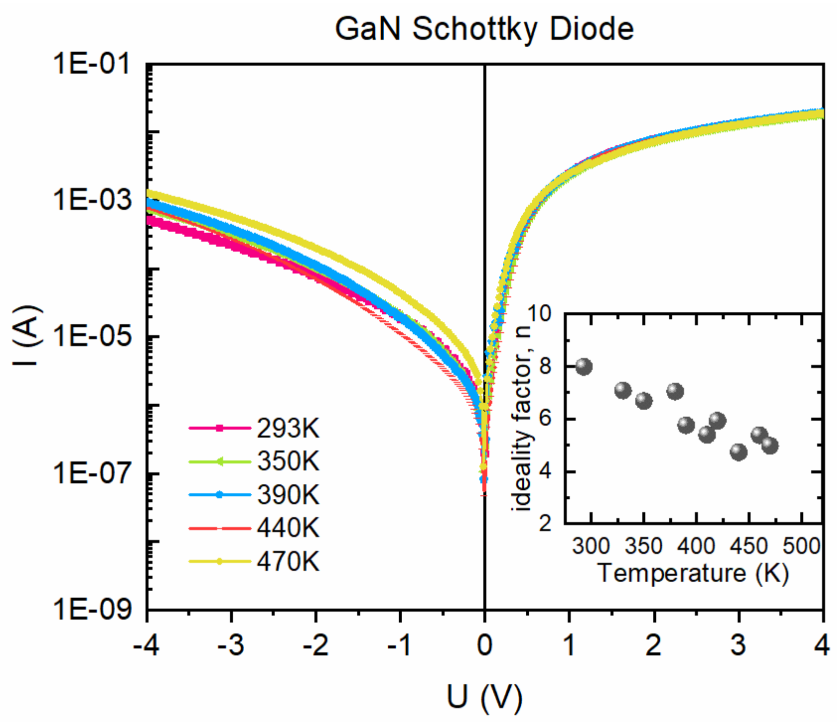


*Figure S1 I-V-T characteristic of reference GaN Schottky Diode. Inset show the temperature dependence of ideality factor, n.*

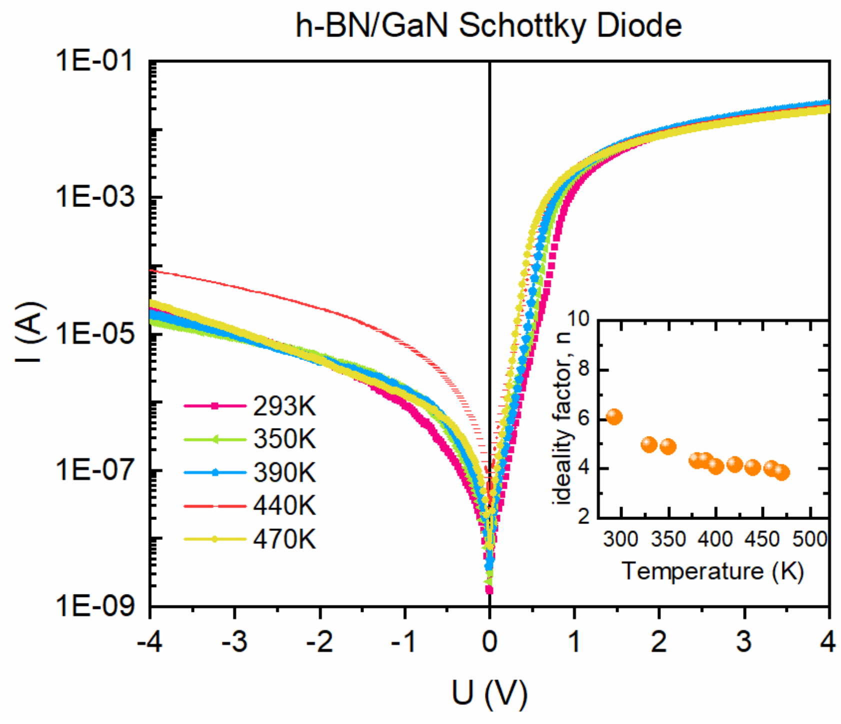


*Figure S2 I-V-T characteristic of h-BN/GaN Schottky Diode. Inset show the temperature dependence of ideality factor, n.*

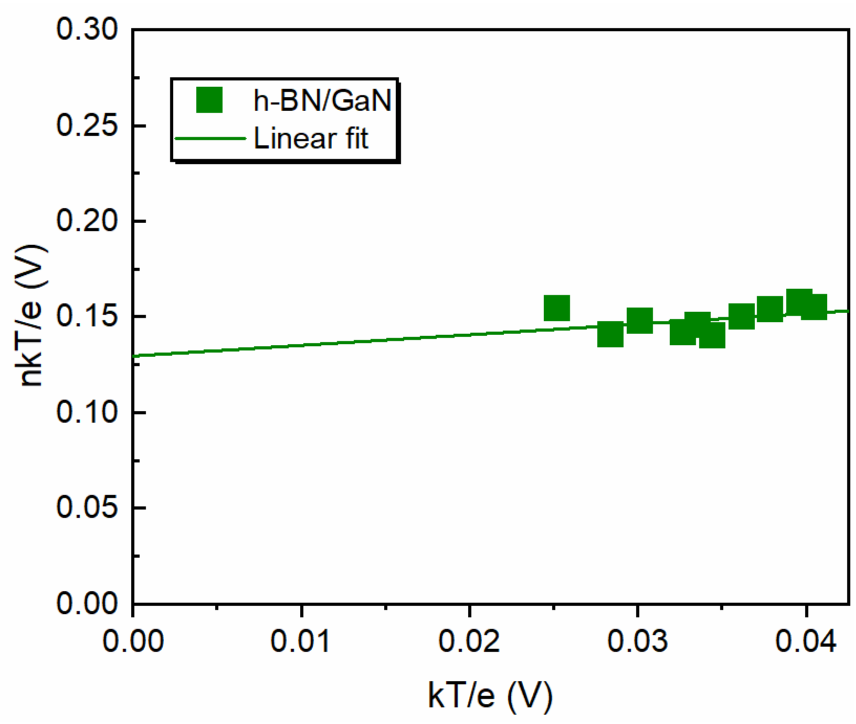


*Figure S3 Plot of nkT/e as a function of kT/e for identifying the current transport mechanism*[1].

The temperature-dependent I-V measurements have been conducted for both kinds of diodes. The shape of I-V curves indicates that Schottky character of the diode was preserved for h-BN containing structure. The ideality factor $n$ was calculated from the slope of the linear region of the I-V forward bias from $n = q/kT(dV/d \ln I)$. The temperature dependence of $n$ for GaN and h-BN/GaN Schottky diodes is shown in the insets of Figures S1 and S2, respectively. The unusually high $n$ values obtained for GaN structure may be related to the fact, that diode was processed on the van Hoof structure, where the top layer is nominally undoped, what is not a standard approach in the Schottky diode fabrication. For GaN and h-BN/GaN diodes ideality factor decreases with increasing temperature. Such a behavior was widely reported in the literature and is attributed to the barrier inhomogeneity at the metal/semiconductor interface.[2–4] According to ref.[1] for V > 2kT/q the I-V relation can be written as $I=I_0exp(qV/nkT)$ and if the ideality factor exhibits the temperature dependence, the current transport mechanism can be identified from $nT$ plotted as a function of $T$. The latter has been checked for h-BN/Schottky diode and is presented in the Figure S3. Observed linear dependence with the intercept >0 stands for thermionic emission as the dominant transport mechanism with $n$ showing the $T_0$ effect.[1] What is more, since tunneling through the barrier is likely to appear for dopant concentration exceeding[1] $10^{17}$ $cm^{-3}$ we do not expect it to be a dominant transport mechanism in our structures since electrical contacts are made on the undoped layer (top layer in van Hoof structure), where

expected carrier concentration is on the level of $10^{16}$ $cm^{-3}$. It is also worth to note that large values of $n$ observed for h-BN/GaN diode are consisted with values reported for other van der Waals/covalent crystal interfaces and can be assigned to the interfacial disorder.[5–9]

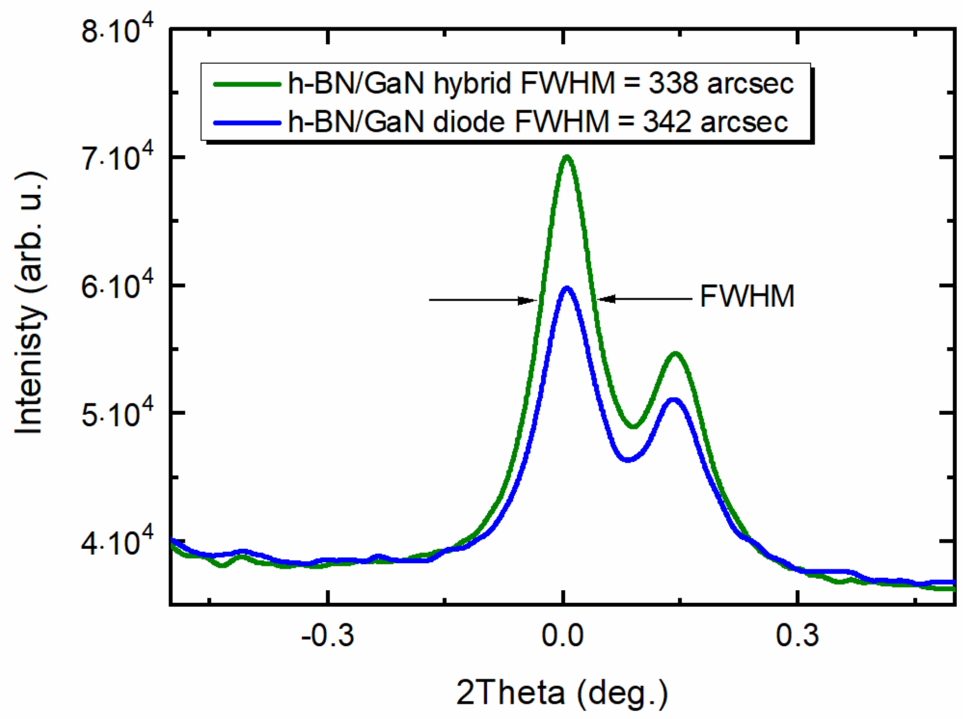


*Figure S4 XRD diffraction spectrum of h-BN (0004) reflection.*

In order to compare the h-BN's quality in h-BN/GaN hybrid and diode the FWHM of h-BN (0004) reflection was measured, since commonly it acts as a basic indicator of structures' quality.[10] The Bragg-Bretano configuration of measurement was used as for the diffraction curves presented in Figure 1 (b) ( main part of the article). In Figure S4 two peaks are visible, since they are Kα1 and Kα2 lines of (0004) reflection, what is typical for this experimental configuration. The obtained values of FWHM were 0.094 (338 arcsec) and 0.095 (342 arcsec) for the hybrid and diode, respectively. This indicates the comparable quality of h-BN present in both structures.

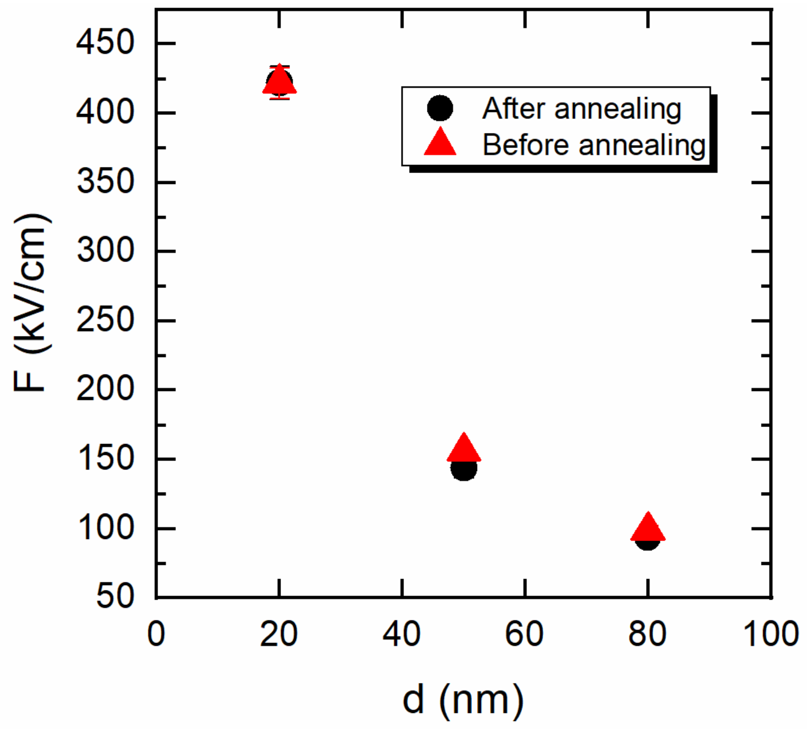


*Figure S5 The comparison of built-in electric field in h-BN/GaN hybrids before and after annealing step during the h-BN transfer process.*

To verify the influence of annealing step during the h-BN transfer on the quality of h-BN/GaN interface, the electric field present in the structures was probed by CER for samples before and after annealing. As it can be seen in Figure S5, no significant alteration takes place. The experimental errors are smaller that points' size.

References:


(1) Sharma, B. L. *Metal-Semiconductor Schottky Barrier Junctions and Their Applications*; Plenum Press: New York, 1984.

(2) Doğan, S.; Duman, S.; Gürbulak, B.; Tüzemen, S.; Morkoç, H. Temperature Variation of Current–Voltage Characteristics of Au/Ni/n-GaN Schottky Diodes. *Phys. E Low-Dimens. Syst. Nanostructures* **2009**, *41* (4), 646–651. https://doi.org/10.1016/j.physe.2008.10.020.

(3) Hajjiah, A.; Alkhabbaz, A.; Badran, H.; Gordon, I. The Effect of Temperature on the Forward Bias Electrical Characteristics of Both Pure Ni and Oxidized Ni/Au Schottky Contacts on n-Type GaN: A Case Study. *Results Phys.* **2020**, *19*, 103656. https://doi.org/10.1016/j.rinp.2020.103656.

(4) Werner, J. H.; Güttler, H. H. Barrier Inhomogeneities at Schottky Contacts. *J. Appl. Phys.* **1991**, *69* (3), 1522–1533. https://doi.org/10.1063/1.347243.

(5) Moun, M.; Kumar, M.; Garg, M.; Pathak, R.; Singh, R. Understanding of MoS2/GaN Heterojunction Diode and Its Photodetection Properties. *Sci. Rep.* **2018**, *8* (1), 11799. https://doi.org/10.1038/s41598-018-30237-8.

(6) Moun, M.; Singh, R. Exploring Conduction Mechanism and Photoresponse in *P*- GaN /*n*- $MoS_2$ Heterojunction Diode. *J. Appl. Phys.* **2020**, *127* (13), 135702. https://doi.org/10.1063/1.5143015.
(7) Zhang, K.; Jariwala, B.; Li, J.; Briggs, N. C.; Wang, B.; Ruzmetov, D.; Burke, R. A.; Lerach, J. O.; Ivanov, T. G.; Haque, M.; Feenstra, R. M.; Robinson, J. A. Large Scale 2D/3D Hybrids Based on Gallium Nitride and Transition Metal Dichalcogenides. *Nanoscale* **2018**, *10* (1), 336–341. https://doi.org/10.1039/C7NR07586C.
(8) Ruzmetov, D.; Zhang, K.; Stan, G.; Kalanyan, B.; Bhimanapati, G. R.; Eichfeld, S. M.; Burke, R. A.; Shah, P. B.; O'Regan, T. P.; Crowne, F. J.; Birdwell, A. G.; Robinson, J. A.; Davydov, A. V.; Ivanov, T. G. Vertical 2D/3D Semiconductor Heterostructures Based on Epitaxial Molybdenum Disulfide and Gallium Nitride. *ACS Nano* **2016**, *10* (3), 3580–3588. https://doi.org/10.1021/acsnano.5b08008.
(9) Acar, M.; Mobtakeri, S.; Ertugrul, M.; Gur, E. Fabrication and Analysis Of 2D/3D Heterojunction Between Continuous Few-Layer WS2 Film and Si (100). *Hittite J. Sci. Eng.* **2021**, *8* (1), 01–05. https://doi.org/10.17350/HJSE19030000206.
(10) Moram, M. A.; Vickers, M. E. X-Ray Diffraction of III-Nitrides. *Rep. Prog. Phys.* **2009**, *72* (3), 036502. https://doi.org/10.1088/0034-4885/72/3/036502.
